# Determinants of Postnatal Care and Timing of the First Postnatal Care for Newborns in Ethiopia: Further Analysis of 2019 Ethiopian Demographic and Health Survey

**DOI:** 10.3389/fped.2022.809643

**Published:** 2022-03-24

**Authors:** Sewnet Adem Kebede, Adisu Birhanu Weldesenbet, Biruk Shalmeno Tusa

**Affiliations:** ^1^Department of Epidemiology and Biostatistics, Institute of Public Health, College of Medicine and Health Sciences, University of Gondar, Gondar, Ethiopia; ^2^Department of Epidemiology and Biostatistics, College of Health and Medical Sciences, Haramaya University, Haramaya, Ethiopia

**Keywords:** PNC, determinant, Ethiopia, postnatal period, newborn

## Abstract

**Background:**

Neonatal mortality remains a persisting public health challenge in Ethiopia. Most of the factors that lead to neonatal deaths could be prevented through postnatal checkups. However, in Ethiopia, the provision of postnatal care (PNC) continues to be low. This study aims to assess the socioeconomic and demographic factors associated with PNC visits and the timing of PNC among newborns in Ethiopia.

**Methods:**

Using the Ethiopia Mini Demographic and Health Survey (EMDHS) 2019, a total weighted sample of 2,105 women aged 15–49 giving birth in the 2 years preceding the survey were included in the study. The generalized linear mixed models were separately fitted to identify factors associated with any PNC for newborns delivered at home and health facilities. Multinomial logistic regression was used to assess the timing of PNC with their associated factors.

**Results:**

Overall, only 13% (95% CI: 11.2, 14.0) of the newborns received PNC in Ethiopia. Among newborns delivered at home, utilization of any PNC was determined by region, maternal educational status, and birth order. On the other hand, among newborns delivered in a health facility, region, number of antenatal care (ANC) visits, and religion were determinants of any PNC. Furthermore, utilization of the first PNC within 48 h after the delivery was determined by region and religion. On the other hand, utilization of the first PNC after 48 h after the delivery was determined by region number of ANC visits, maternal educational status, and religion.

**Conclusion:**

The finding of the current study revealed low coverage of PNC among newborns regardless of the place of delivery in Ethiopia. The study makes the following recommendation: increase community health education on PNC, encourage delivery at health facilities, and link community home birth with PNC. It will be more valuable if there is sharing good practice.

## Introduction

Postnatal care (PNC) is the care given to the mother and her newborn baby immediately after the birth and extends up to 6 weeks (42 days) after birth (1, 2). PNC is important to reduce death, disability, and missed opportunities to promote healthy behaviors in women, newborns, and children (3). Globally, 2.4 million children died in the first month of life in 2019 (4). Every year in Africa, at least 870,000 newborns die in the first week after birth (3). In 2019, Sub-Saharan Africa had the highest neonatal mortality rate at 27 deaths per 1,000 live births (5, 6). Ethiopia is ranked fourth among the top 10 countries with the highest number of newborn deaths in 2019 (6). According to the most recent Ethiopia Mini Demographic and Health Survey (EMDHS), the early newborn death rate was very high, with 30 babies dying in the first 28 days of life for every 1,000 live births (7).

The most vulnerable time for both mother and newborn is during the first 6 weeks after birth (postnatal period). Health checks during this time especially the first 2 days after delivery are essential (3, 8). Approximately 7,000 newborns die every day, with about a third of all neonatal deaths occurring within the first day after birth and close to three-quarters occurring within the first week of life (9, 10).

Elsewhere, evidence shows that most of the factors that lead to neonatal deaths could be prevented through postnatal checkups (8, 11–13). PNC for the baby is an important opportunity to check for danger signs, such as insufficient feeding, fast breathing, severe chest in-drawing, lethargy, fever, low body temperature, or jaundice. Simultaneously, mothers can receive advice on how to identify and respond to these symptoms, as well as the benefits of exclusive breastfeeding and immunization (8).

The WHO recommends PNC within 24 h of birth, regardless of where the baby is born. Newborns should receive at least three additional PNC visits by a skilled provider, on day 3 (48–72 h after birth), between day 7 and day 14, and again 6 weeks after birth (14). Globally, only 48% of newborns receive a postnatal health check within the recommended time period (8). PNC programs are among the weakest of all reproductive and child health programs in the Africa region (3). In Ethiopia, as in all countries, the postnatal period is often marked by specific cultural practices and socioeconomic factors, and mothers and newborns spend most of this period at home (1, 3, 15).

The 2019 EMDHS shows some improvement in the survival rates of infants and children under age 5 in recent years. According to the EMDHS, the under-five mortality rate dropped from 123 to 43 deaths per 1,000 live births between 2005 and 2019, and neonatal mortality decreased from 39 to 29 deaths per 1,000 live births between 2005 and 2016 but has remained stable since 2016. This situation may be explained in part by the low level of PNC (34%) (7).

As mortality among children under five declines globally, deaths among these children are more and more concentrated in the first days of life. This makes focus on newborn care more critical than ever before (8). The Ethiopian government proposed the National Newborn and Child Survival Strategy (2015–2020), which aims to reduce the neonatal mortality rate from 28/1,000 live births to 11/1,000 live births (16). If all newborns received high-impact and cost-effective interventions during the postnatal period, it is estimated that neonatal mortality could be reduced to 12 per 1,000 live births. High PNC coverage could save up to 210,234 newborn lives a year in Ethiopia (16) and help the country to meet the Sustainable Development Goal of ending preventable deaths of newborns and children under 5 years of age by the year 2030 (17).

To increase coverage of PNC of newborns in Ethiopia, a better understanding of its socioeconomic and demographic factors is important. The objective of this study was to assess socioeconomic and demographic factors associated with any PNC for newborns and the timing of the first PNC. Understanding such factors may help develop necessary strategies and interventions to help improve not only PNC coverage but also its timing in the most critical period (within 48 h) and, in turn, increase neonatal survival chances in Ethiopia.

## Methods

### Study Design, Period, and Setting

A community-based cross-sectional study was employed in Ethiopia. The survey was conducted from March 21 to June 28, 2019, based on a nationally representative sample that provides estimates at the national and regional levels. Ethiopia is situated in the Horn of Africa with nine regional states (Tigray, Afar, Amhara, Oromia, Somalia, Benishangul-Gumuz, Southern Nations, Nationalities and People's Region (SNNPR), Gambella, and Harari) and two city administrative councils (Addis Ababa and Dire Dawa) of administrative boundaries (7).

### Study Population, Sample Size, and Sampling Procedure

**Figure 1:**
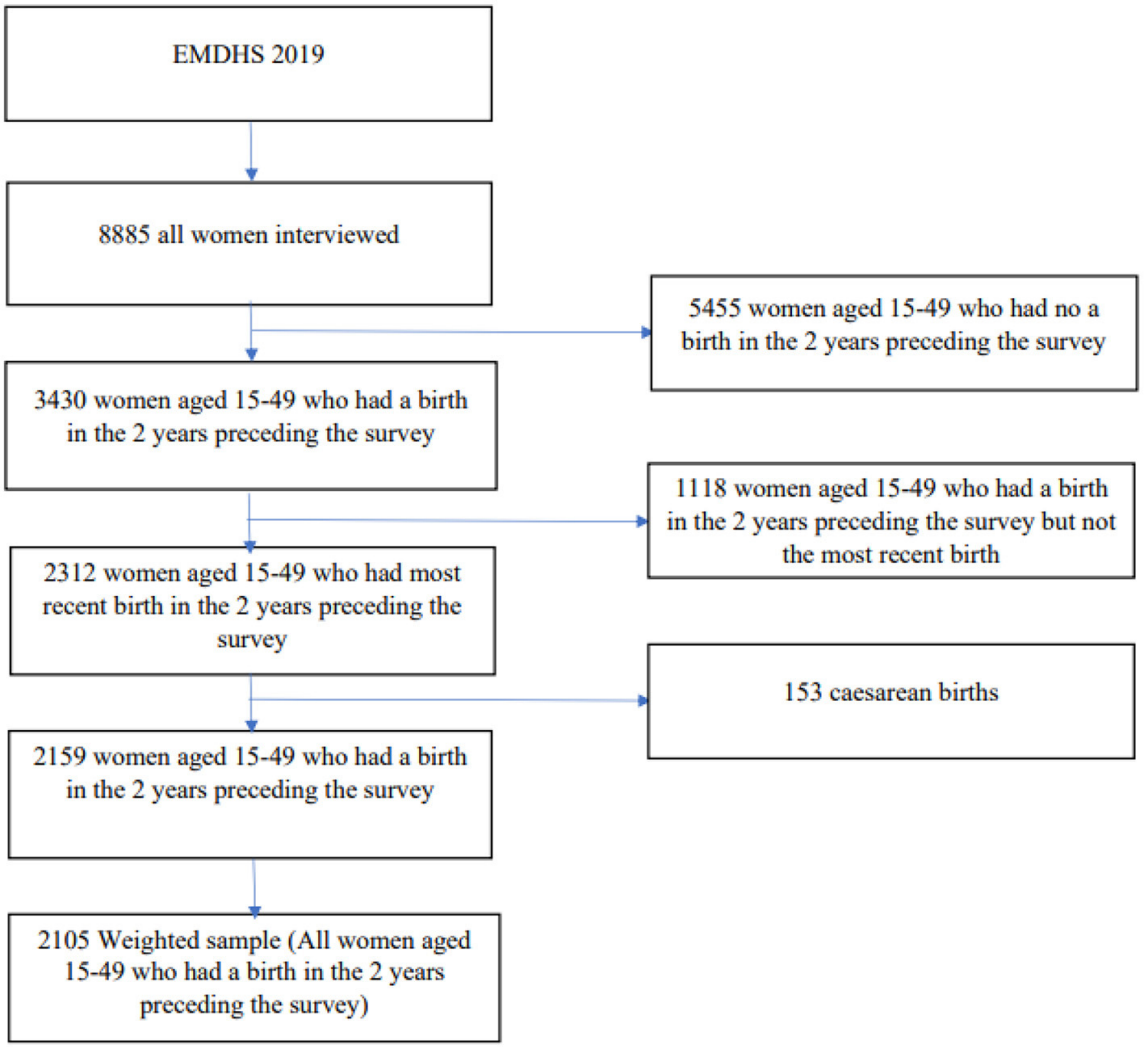
Final sample size and schematic presentation of how the study sample was selected.

All women aged 15–49 who had a birth in the 2 years preceding the survey in the selected enumeration areas (EAs) were the study population. One hundred fifty-three (153) cesarean births were excluded from the final analysis because they were more likely to receive PNC independent of the mothers' demographic or socioeconomic status. A total weighted sample of 2,105 births was included in the study (Figure 1). Of these, 942 were delivered at home, and 1,163 were delivered in a health facility. The 2019 EMDHS sample was stratified and selected in two stages. Each region was stratified into urban and rural areas, yielding 21 sampling strata. To ensure that survey precision is comparable across regions, the sample allocation has been done through an equal allocation where 25 EAs are selected from eight regions. However, from the three larger regions Amhara, Oromia, and SNNPR, 35 EAs for each were selected. In the first stage, a total of 305 EAs (93 in urban areas and 212 in rural areas) were selected with probability proportional to EA size. In the second stage of selection, a fixed number of 30 households per cluster were selected with an equal probability of systematic selection from the newly created household listing (7).

### Data Collection Procedure and Variables

After permission was granted through an online request by explaining the objective of our study, the data were accessed from the DHS program official database www.meauredhs.com. The EMDHS 2019 Individual Women's records (IR) were used, and the dependent variable with its important predictors was extracted.

The outcome variables were any postnatal checkup within 6 weeks after delivery “0 = No PNC; and 1 = Yes any PNC” and timing of first PNC “0 = No PNC,” “1 > 48 h,” and “2 ≤ 48 h” (18). The independent variables were maternal age, region, residence, wealth index, number of antenatal care (ANC) visits, maternal educational status, religion, birth order, sex of household head, and current marital status.

### Statistical Analysis

The analysis was done using Stata 14 software. In EDHS data, women within a cluster may be more similar to each other than women in the rest of the country. This goes against the regression model's assumptions of observation independence and equal variance across clusters. This implies the need to consider the between-cluster variability using advanced models. As a result, a mixed model with both fixed and random effects was used. Logistic regression and generalized linear mixed models (GLMMs) were fitted because the outcome variable was binary. Model comparison was done based on Akaike information criteria (AICs), Bayesian information criteria (BICs), and deviance value. Because it had the lowest AIC, the GLMM was chosen. Two GLMMs were fitted to identify factors associated with any PNC for newborns: one for newborns delivered at home and the other for those delivered in a health facility. Adjusted odds ratio (AOR) with 95% CI were reported, and those variables with *p* < 0.05 were considered as statistically significant factors associated with any PNC for newborns. Additionally, a multinomial logistic regression was used to assess the timing of PNC with their associated factors. In both models, “never received any PNC” was the reference category.

## Results

### Characteristics of Study Participants According to Place of Delivery, Ethiopia Mini Demographic and Health Survey 2019

The total sample for the analysis was 2,105 newborns of whom 1,163 (55.26%) were delivered at health facilities while 942 (44.74%) were delivered at home. Newborns delivered at home had a higher proportion of being 4th-order births (55.74%) than newborns delivered in a health facility (30.86%). For both home and facility births, about one-third of the mothers were aged 25–29 at the time of birth. The proportion of newborns delivered at home was higher in the Oromia and Amhara regional states of Ethiopia. The proportion of delivering at home decreased with an increased wealth index (Table 1).

**Table 1:** Characteristics of study participants according to the place of delivery in Ethiopia, 2019.

**Variables**	**Home delivery**		**Facility delivery**		**Total**	
	**%**	***n***	**%**	***n***	**%**	***n***
**Maternal age**						
15–19	8.18	77	8.86	103	8.56	180
20–24	19.05	179	27.72	322	23.84	502
25–29	31.52	297	29.28	340	30.28	637
30–34	20.74	195	17.52	204	18.96	399
35–39	13.29	125	11.66	136	12.39	261
40–44	6.58	62	4.29	50	5.31	112
45–49	0.65	6	0.66	8	0.66	14
**Region**						
Tigray	3.84	36	10.24	119	7.38	155
Afar	2.21	21	0.91	11	1.49	32
Amhara	18.52	174	22.22	259	20.56	433
Oromia	43.31	408	35.86	417	39.19	825
Somali	10.13	95	3.17	37	6.28	132
Benishangul-Gumuz	0.75	7	1.47	17	1.15	24
SNNPR	20.25	191	18.98	221	19.55	412
Gambella	0.24	2	0.67	8	0.48	10
Harari	0.18	2	0.36	4	0.28	6
Addis Ababa	0.24	3	5.31	61	3.04	64
Dire Dawa	0.33	3	0.81	9	0.60	12
**Residence**						
Urban	14.1	133	36.13	420	26.27	553
Rural	85.9	809	63.87	743	73.73	1,552
**Number of ANC visit**						
<4	95.61	900	93.94	1,093	94.69	1,993
≥4	4.39	41	6.06	70	5.31	112
**Maternal educational status**						
No education	63.77	600	32.42	377	46.44	978
Primary	34.42	324	44.38	516	39.92	840
Secondary	1.15	11	14.72	171	8.65	182
Higher	0.66	6	8.48	99	4.98	105
**Religion**						
Orthodox	28.33	267	41.77	486	35.76	753
Protestant	30.95	291	23.43	273	26.79	564
Muslim	38.44	362	34.06	396	36.02	758
Traditional	2.28	21	0.74	9	1.43	30
**Birth order**						
First	12.35	116	32.99	384	23.75	450
Second	17.06	161	23.43	272	20.58	433
Third	14.85	140	12.73	148	13.68	288
Fourth and more	55.74	525	30.86	359	41.99	884
**Wealth index**						
Poorest	38.2	360	8.63	100	21.86	460
Poorer	25.18	237	18.27	212	21.36	449
Middle	20.05	189	17.51	204	18.65	393
Richer	11.85	112	21.72	253	17.30	365
Richest	4.73	45	33.87	393	20.83	438
**Current marital status**						
Never in union	0.61	6	0.86	10	0.75	16
Married	94.88	893	95.16	1,107	95.03	2,000
Divorced	4.51	43	3.97	46	4.21	89

*SNNPR, Southern Nations, Nationalities and People's Region; ANC, antenatal care*.

### Postnatal Care by Place of Delivery and Timing of the First Postnatal Care

**Figure 2:**
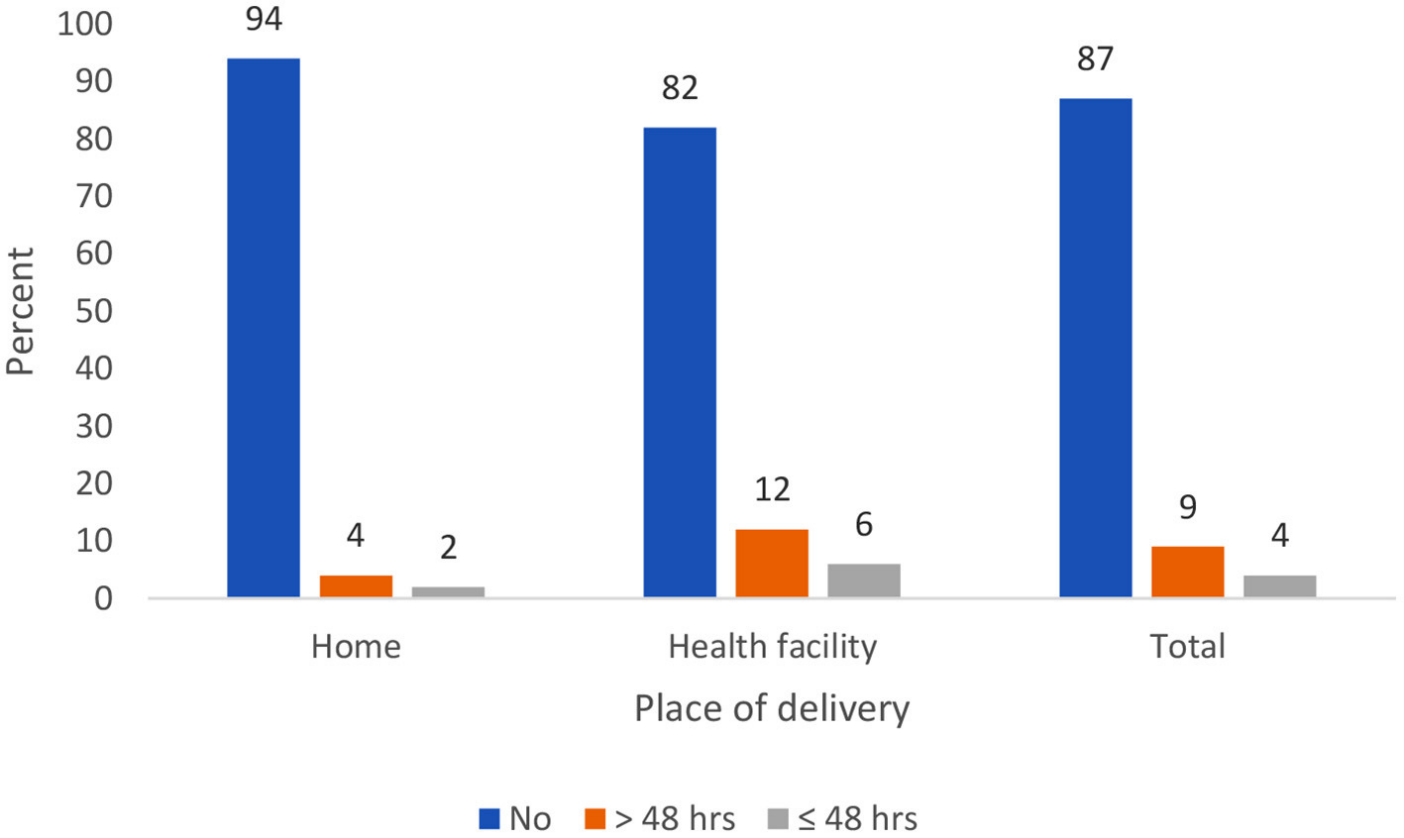
Percent distribution of newborns by timing of the first postnatal care.

Overall, only 13% (95% CI: 11.2, 14.0) of the newborns received PNC after birth in Ethiopia. About the place of delivery, 18% of newborns delivered in a health facility received PNC as compared with 6% newborns delivered at home. The majority of newborns received their first PNC after 2 days for both home and health facility delivery (Figure 2).

### Determinants of Postnatal Care for Newborns

In home delivery, the odds of having a PNC visit were 2.79 times higher for newborns whose mothers completed primary education as compared to those whose mothers had no education. The odds of home newborns having a PNC visit were 65, 62, and 61% lower for newborns whose mother had a first-order child, second-order child, and third-order child, respectively, as compared to those who had a fourth- or higher-order child.

Among newborns delivered in a health facility, the odds of receiving any PNC for newborns whose mothers had (4+ ANC) visits during pregnancy had 97% higher odds as compared to newborns whose mothers had fewer than four ANC visits. Newborns whose mother was Protestant had 63% lower odds of receiving any PNC as compared to those whose mother was Orthodox (Table 2).

**Table 2:** Multivariable mixed-effect logistic regression analysis of any postnatal care among newborns by place of delivery in Ethiopia, 2019.

**Variables**	**Home delivery**	**Facility delivery**
	**AOR (95% CI)**	**AOR (95% CI)**
**Maternal age**		
15–24	1	1
25–34	0.93 (0.39, 2.21)	0.93 (0.59, 1.48)
35+	0.91 (0.31, 2.76)	1.08 (0.55, 2.15)
**Region**		
Tigray	2.73 (0.41, 18.55)	5.14 (0.99, 26.4)
Afar	1	1
Amhara	1.31 (0.20, 8.44)	3.86 (0.73, 20.1)
Oromia	**0.19 (0.38, 0.99)**	**8.61 (1.73, 42.7)**
Somali	0.39 (0.11, 1.49)	2.32 (0.33, 16.2)
Benishangul-Gumuz	0.61 (0.11, 3.26)	3.28 (0.64, 16.9)
SNNPR	0.49 (0.08, 2.96)	**11.7 (2.30, 60.3)**
Gambella	1.39 (0.26, 7.48)	4.78 (0.86, 26.3)
Harari	1.40 (0.31, 6.41)	**6.13 (1.24, 30.1)**
Addis Ababa	5.96 (0.21, 16.6)	**5.89 (1.17, 29.6)**
Dire Dawa	3.02 (0.76, 11.9)	**5.98 (1.21, 29.5)**
**Residence**		
Urban	1	1
Rural	1.11 (0.25, 4.83)	1.04 (0.57, 1.89)
**Number of ANC visit**		
<4	1	1
≥4	0.59 (0.14, 2.49)	**1.97 (1.01, 4.26)**
**Maternal educational status**		
No education	1	1
Primary	**2.79 (1.41, 5.05)**	1.06 (0.67, 1, 67)
Secondary	3.17 (0.67, 14.9)	0.93 (0.49, 1.73)
Higher	9.18 (0.48, 17.5)	1, 47 (0.75, 2.87)
**Religion**		
Orthodox	1	1
Protestant	0.72 (0.18, 2.85)	**0.37 (0.19, 0.71)**
Muslim	0.82 (0.19, 2.31)	0.67 (0.41, 1.08)
**Birth order**		
First	**0.35 (0.11, 1.00)**	1.15 (0.63, 2.14)
Second	**0.38 (0.15, 0.99)**	1.34 (0.77, 2.33)
Third	**0.39 (0.16, 0.98)**	1.18 (0.66, 2.12)
Fourth and more	1	1
**Wealth index**		
Poorest	1	1
Poorer	1.32 (0.62, 2.84)	1.45 (0.67, 3.12)
Middle	1.40 (0.58, 3.37)	1.37 (0.62, 3.01)
Richer	1.25 (0.43, 3.58)	0.97 (0.44, 2.13)
Richest	1.43 (0.32, 6.31)	1.42 (0.60, 3.35)
**Current marital status**		
Never in union	0.63 (0.03, 11.9)	3.61 (0.65, 20.1)
Married	0.67 (0.19, 2.31)	1.80 (0.73, 4.45)
Divorced	1	1
**Sex of household head**		
Male	1	1
Female	0.77 (0.33, 1.78)	1.36 (0.87, 2.13)

*ANC, antenatal care; AOR, adjusted odds ratio; SNNPR, Southern Nations, Nationalities and People's Region. Bold value means P-value ≤ 0.05*.

### Factors Associated With Timing of the First Postnatal Care

Newborns from the SNNPR region were 4.24 times more likely [AOR = 4.24; 95% CI: 1.47–12.2] to go through the first PNC within 48 h after delivery than newborns from the Afar region. Newborns whose mothers were Protestant were 62% less likely [AOR = 0.38; 95% CI: 0.17–0.85] to go through the first PNC within 48 h after delivery than newborns whose mother was Orthodox.

Newborns whose mothers made (4+ ANC) visits were 91% more likely [AOR = 1.91; 95% CI: 1.07–3.79] to go through the first PNC after 48 h of delivery than newborns whose mothers made fewer than four ANC visits. Newborns to mothers who had primary education were 51% more likely [AOR = 1.51; 95% CI: 1.01–2.30] to go through the first PNC after 48 h of delivery than newborns whose mothers had no education, and newborns to mothers who had higher education were 2.21 times more likely [AOR = 2.21; 95% CI: 1.15–4.24] to go through the first PNC after 48 h of delivery than newborns whose mothers had no education at all. Newborns whose mothers were Protestant were 58% less likely [AOR = 0.42; 95% CI: 0.23–0.79] to go through the first PNC after 48 h of delivery than newborns whose mother was Orthodox (Table 3).

**Table 3:** Factors associated with timing of the first postnatal checkups among newborns in Ethiopia, 2019.

**Variable**	**Within 48 h**	**After 48 h**
	**AOR (95% CI)**	**AOR (95% CI)**
**Maternal age**		
15–24	1	1
25–34	1.22 (0.63, 2.35)	0.91 (0.58, 1.41)
35+	1.13 (0.45, 2.77)	1.08 (0.57, 2.06)
**Region**		
Tigray	2.37 (0.75, 7.52)	**3.82 (1.26, 11.5)**
Afar	1	1
Amhara	1.07 (0.31, 3.76)	**3.44 (1.15, 10.3)**
Oromia	1.17 (0.38, 3.59)	**3.64 (1.29, 10.2)**
Somali	0.58 (0.17, 2.06)	0.93 (0.24, 3.53)
Benishangul-Gumuz	1.69 (0.58, 4.92)	1.54 (0.46, 5.08)
SNNPR	**4.24 (1.47, 12.2)**	**3.72 (1.21, 11.4)**
Gambella	1.60 (0.45, 5.69)	**4.12 (1.31, 12.9)**
Harari	0.75 (0.21, 2.72)	**6.21 (2.24, 17.2)**
Addis Ababa	0.21 (0, 02, 1.94)	**7.82 (2.62, 23.4)**
Dire Dawa	1.27 (0.39, 4.06)	**6.28 (2.27, 17.4)**
**Residence**		
Urban	1	1
Rural	0.74 (0.41, 1.35)	1.12 (0.71, 1.78)
**Number of ANC visit**		
<4	1	1
≥4	0.87 (0.26, 2.87)	**1.91 (1.07, 3.79)**
**Maternal educational status**		
No education	1	1
Primary	1.49 (0.87, 2.56)	**1.51 (1.01, 2.30)**
Secondary	1.16 (0.46, 2.91)	1.53 (0.84, 2.77)
Higher	2.04 (0.75, 5.47)	**2.21 (1.15, 4, 24)**
**Religion**		
Orthodox	1	1
Protestant	**0.38 (0.17, 0.85)**	**0.42 (0.23, 0.79)**
Muslim	0.74 (0.37, 1.48)	0.74 (0.46, 1.19)
**Birth order**		
First	0.53 (0.26, 1.07)	1.11 (0.62, 1.98)
Second	0.92 (0.49, 1.69)	0.98 (0.58, 1.67)
Third	0.75 (0.38, 1.47)	0.87 (0.50, 1.52)
Fourth and more	1	1
**Current marital status**		
Never in union	2.08 (0.19, 23.1)	1.91 (0.44, 8.35)
Married	1.54 (0.45, 5.31)	1.10 (0.51, 2.36)
Divorced	1	1
**Sex of household head**		
Male	1	1
Female	1.72 (0.96, 3.07)	1.04 (0.67, 1.62)
**Place of delivery**		
Home	1	1
Health facility	1.19 (0.70, 2.02)	**1.67 (1.11, 2.53)**

*ANC, antenatal care; AOR, adjusted odds ratio. Bold value means P-value ≤ 0.05*.

## Discussion

PNC appears to be a component of maternal and child care service that is poorly utilized despite being provided at a critical period for the survival of both mother and baby. This study aimed to identify factors associated with any PNC for newborns and also factors associated with the timing of the first PNC among newborns by place of delivery in Ethiopia.

The finding showed that the overall coverage of PNC for newborns was only 13% in Ethiopia. This finding is lower than that of a study done in India (69.1%) (19), Gambia (26.7) (20), and Bangladesh (33%) (21). This might be due to study time, study population, and sample size variation. Furthermore, cultural practice variation across the countries during the postnatal period can be the reason.

About the place of delivery, newborns delivered at home were less likely than those delivered at a health facility to receive PNC (6 vs. 18%). This finding is consistent with that of other studies done in Ethiopia (22), Zambia (23), Nigeria (24), and Nepal (25). This might be because women who gave birth in a health facility have greater opportunity for health education related to PNC services at the time of delivery and thus have access to learning about the types, benefits, and availabilities of PNC services during their stay in the health facility (26). Additionally, women who gave birth at home are part of more traditional practices and are consequently less likely to use PNC services.

Region was a significant predictor of PNC visits among newborns in this study. This finding is consistent with that of similar studies done in Ethiopia (27, 28). The possible justification could be the differences in health service accessibility and quality of healthcare between regions. In addition, it could be the result of sociocultural differences during postnatal period activities.

This study revealed that maternal education was found to be a predictor of PNC visits. This finding is consistent with that of previous studies on Rwanda (29), Pakistan (30), Nepal (31), and Nigeria (24). The possible explanation for this similarity could be due to the fact that education helps raise mothers' awareness and acceptance of new ideas, as well as give better information to other women about the use of PNC services for both mothers and newborns.

The number of ANC visits is an important determinant of PNC utilization. This evidence coincides with that of previous studies elsewhere (31–33). One probable explanation is that ANC provides a chance for health promotion. As a result, women who utilize ANC have appropriate counseling as well as greater knowledge on the advantages of PNC during the ANC session. Additionally, women may make birth plans in collaboration with their ANC provider, which will boost the utilization of hospital delivery and postnatal services (34).

Notably, this study found that newborns whose mother was Protestant had lower odds of receiving any PNC compared to those whose mother was Orthodox. Similar findings were reported in Ethiopia (35). Our study also reveals that newborns whose mother was Protestant were associated with lower odds of receiving PNC either within 48 h or after 48 h (62 vs. 58%). Even though we were unable to locate any documentation to explain our findings, the possible explanation could be the differences in religious advice among followers to use the PNC service and religious leaders' participation in health-related aspects. However, more research is needed to investigate the disparities in PNC utilization among different religious groups.

The birth order of the child showed a significant effect on PNC for newborns delivered at home. The findings of this study are opposite to those of studies conducted in India (19), Pakistan (30), and Ethiopia (36). This is possibly related to the maternal experience of childbirth. Since the association between birth order and PNC visits for the newborn is controversial, it needs further research to determine whether this birth order difference contributes to increasing PNC utilization.

### Limitation of the Study

This study is not free from limitations. First, some potentially important cultural factors following birth that may affect PNC care were not assessed since they are not available in DHS data. Second, since EMDHS 2019 is a mini-version, some important factors that may affect both utilization and timing of first PNC were not collected like media exposure, current pregnancy wanted, occupational status, and healthcare decision-making autonomy. Another limitation of this study is recall bias, as EDHS was a questionnaire-based survey and relied on the respondents' memories of events that occurred 2 years before the survey.

## Conclusion

Low coverage of PNC among newborns was found regardless of the place of delivery in Ethiopia. This study also demonstrates that various factors influence the utilization and the timing of PNC for newborns. To increase PNC coverage, it is better to increase community health education on PNC and health facility delivery. To do so, healthcare providers should use ANC visits as a mechanism to counsel and provide information about the benefit and availability of PNC and encourage delivery at a health facility. It is better to develop and strengthen community-based service strategies to link community home birth with PNC since PNC coverage among newborns delivered at home is very low. We also recommend sharing experiences from regions that have better PNC coverage.

## Data Availability Statement

The original contributions presented in the study are included in the article/supplementary files, further inquiries can be directed to the corresponding author.

## Ethics Statement

The study does not involve the collection of information from subjects. Consent to participate is not applicable. We sent a one-page proposal abstract of the study to the DHS program office. They gave permission to access the data with reference number 158703.

## Author Contributions

Conception of the work, design of the work, acquisition of data, analysis, and interpretation of data were done by SK. Data curation, drafting the article, revising it critically for intellectual content, validation, and final approval of the version to be published were done by SK, AW, and BT. All authors read and approved the final manuscript.

## Conflict of Interest

The authors declare that the research was conducted in the absence of any commercial or financial relationships that could be construed as a potential conflict of interest.

## Publisher's Note

All claims expressed in this article are solely those of the authors and do not necessarily represent those of their affiliated organizations, or those of the publisher, the editors and the reviewers. Any product that may be evaluated in this article, or claim that may be made by its manufacturer, is not guaranteed or endorsed by the publisher.

## Abbreviations

ANC: antenatal care
AOR: adjusted odds ratio
CSA: Central Statistics Agency
EA: enumeration area
EMDHS: Ethiopia Mini Demographic and Health Survey
PNC: postnatal care
RRR: relative risk ratio
SNNPR: Southern Nation and Nationality and Peoples Region.
